# Adolescents' perceptions of using likes, comments, and other reactions—A qualitative investigation

**DOI:** 10.1111/bjdp.12537

**Published:** 2024-12-15

**Authors:** Gemma Rides, Helen Pote, Dawn Watling

**Affiliations:** ^1^ Department of Psychology, Royal Holloway University of London London UK

**Keywords:** adolescents, impression management, online communication, online support, self‐expression, social media

## Abstract

With the majority of young people using social media as a primary form of communication with friends and family, it is becoming increasingly important to understand how adolescents perceive their own and others' online behaviour. Participants (*N* = 34) aged 11–15 years took part in focus group discussions exploring their perceptions of the communication intentions of using online interpersonal feedback, specifically the use of ‘reactions’ (e.g., likes and comments). A thematic analysis of the transcripts indicated that young people are using social media reactions to (i) form and maintain impressions online, (ii) give, receive, and withhold support from others, and (iii) express themselves and tailor their social media experience. Findings show that adolescents are aware of the online social norms surrounding the use of reactions and how the number of reactions relates to their mood and feelings of self‐worth.

## Graphical abstract

[Correction added on 12 June 2025: Graphical Abstract has been included in this version.]

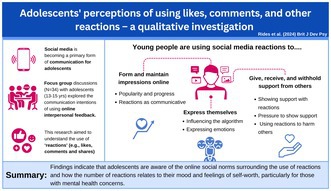

**Statement of contribution**

**What is already known on this subject**

- Online behaviour is important for understanding how social media may link to mental health.
- Reactions (likes and comments) are important to adolescents and have been linked to their wellbeing.

**What the present study adds**

- Social media reactions are used for more than showing they ‘like’ a social media post.
- Reactions are used determine popularity and to give, receive, and withhold social support.
- Adolescents with mental health concerns may worry more about using or receiving reactions.

## INTRODUCTION

The popularity of social media with young people is increasing. In 2023, approximately 93% of 12 to 15‐year‐olds were using social media sites in the United Kingdom (Ofcom, 2023) in comparison to 69% in 2018 (Ofcom, 2019). As these platforms are becoming ubiquitous with adolescents (Kelly et al., 2018), it is crucial that research seeks to further understand the underlying psychological processes behind social media usage. Traditionally, research has focused on the links between time spent on social media and mental health in young people (Vannucci et al., 2017). However, due to the heterogeneity of social media activities, it is vital that research moves beyond screen time and explores how adolescents are using social media, their online behaviours, and how this influences their psychological functioning (Coyne et al., 2020).

Social media platforms vary in terms of the content and site structure. Yet, providing and receiving interpersonal feedback is a core feature of many social media platforms. This interpersonal feedback is typically in the form of reactions. Reactions are tools embedded within social media sites that allow users to engage and interact with the content and communicate with other users. Reactions can be measurable, closed actions, where the user can press a button to ‘like’ or ‘share’ a post, and it is typical that each post will show how many other users have reacted in a particular way (Hayes et al., 2018). Reactions can also be through open text by commenting on social media posts. This function allows the user to use words to share their thoughts or opinions on the content while viewing other users' comments on the post. The main function of reactions is to indicate if a user likes or dislikes content and allows users to communicate their ideas and learn about other users' views. (Tenenboim, 2022).

Reactions can provide support to the social media user through non‐verbal actions (e.g., likes) and verbal actions (e.g., positive comments; Blight et al., 2015). However, adolescents can also intend to use social media reactions to initiate social exclusion, ostracism, and bullying online. This can be achieved by the cyberbullying perpetrator using and not using reactions. A prominent experience that influences individuals to feel excluded or ostracized on social media is when they do not receive the number of reactions they expect on their own posts (Hayes et al., 2018), and this has implications for their self‐esteem, belongingness (Timeo et al., 2020), and negative affect (Reich et al., 2018). A report by NHS Digital (2021) details that approximately 16.7% of 11 to 16‐year‐olds believe that the number of reactions they received had an impact on their mood. Interestingly, research finds that withholding reactions towards others' posts on social media can be purposeful. In qualitative research by Chua and Chang (2016), participants commented on the decision‐making processes around pressing the ‘like’ button on social media, with some withholding the use of the like button so that they do not give too much positive feedback to others. This is perceived to result in the individual experiencing definite, quantifiable rejections or validation from their peers (Winstone et al., 2023). In addition, using reactions, in particular comments, is a common approach that perpetrators use to target victims of cyberbullying (Whittaker & Kowalski, 2015).

Adolescence is a developmental stage that involves exploring self‐identities (Erikson, 1968) and learning to manage the impressions that others form (Aloise‐Young, 1993; Watling & Banerjee, 2007, 2012). The dual factor model proposed by Nadkarni and Hofmann (2012) highlights that social media helps facilitate impression management and self‐presentation by allowing the user to carefully curate an online image by developing a profile, posting content, and receiving interpersonal feedback from others. Indeed, it has been highlighted that adolescents' behaviours on social media have been associated with self‐presentation. The flexibility of social media allows adolescents to engage in identity exploration, where they may portray different selves for different contexts (Michikyan et al., 2015). Reactions play an important role in this identity development, as the type of self that is presented will receive interpersonal feedback from others in the form of reactions.

It is understood that peers become more important as children transition to adolescence, with young people being more attentive to social evaluation (Watling & Banerjee, 2007) and social evaluation concerns being a prominent motive for social comparison to peers (Pomerantz et al., 1995). Due to the breadth of information available on social media and interpersonal feedback on social media being consistent across platforms, there are many opportunities for users to evaluate the self through social comparison (Festinger, 1954; Vogel et al., 2014). Research indicates that the ability to use reactions encourages young people to use downward social comparisons (comparisons to individuals perceived as inferior), which are associated with positive emotions (e.g., happiness), while receiving fewer reactions is more likely to result in the use of upward social comparisons (comparisons to individuals perceived as superior), which are associated with depressive symptoms (Nesi & Prinstein, 2015) and body dissatisfaction (Kelly et al., 2018).

It also appears that adolescents are comparing the number of reactions to previous content that they have shared online. Receiving fewer reactions than expected is thought to be a sign of disapproval from peers and relates to feelings of self‐worth (Chua & Chang, 2016), whereas receiving a greater number of reactions has been linked to higher self‐esteem (Burrow & Rainone, 2017) and to reductions in feelings of loneliness (Yang, 2016). The number of reactions received has been suggested to be connected to perceptions of social approval (Hayes et al., 2018). Adolescents use specific strategies to maximize the number of reactions on a post (Yau & Reich, 2019) and use the number of likes to show superiority over others (Bell, 2019). These findings show the importance of further exploring adolescents' perception of using reactions on social media.

### Research focus

Previous research has indicated that online interpersonal feedback, that is facilitated through social media reactions, appears to be linked to both adolescent mental health outcomes and identity formation. While research has found that the use of reactions is associated with positive and negative experiences online, it does not explicitly explore the intentions behind using reactions. When we consider the possible mental health implications associated with reactions, it is important that researchers explore adolescents' interpretation of reactions as interpersonal feedback and how this might influence their thoughts and feelings. Schønning et al. (2020) highlight that there is limited research that examines adolescent perceptions of the links between social media and feelings of self; they call for more qualitative studies with young people to gain a deeper understanding of the links between social media and mental health.

The present study used an exploratory focus group design to understand how adolescents perceive their own online behaviour, focusing on understanding adolescents' intentions when offering interpersonal feedback, specifically when they do and do not use reactions on social media. The study also sought to consider adolescents' perceptions of interpersonal feedback offered by peers when others use a reaction or not in response to their social media content and how this influences their psychological functioning. Gaining an in‐depth understanding of how adolescents engage with and interpret social media reactions will support the design and development of social media apps to benefit young people and will inform education on adolescent social media use.

## METHODS

### Participants

Thirty‐four adolescents (*M* = 13.56 years, SD = 1.26, Range = 11–15 years; Male = 19, Female = 14, Prefer not to say = 1) were recruited from one school in the UK. Participants identified as white (*n* = 31), mixed or multiple ethnic groups (*n* = 2), and Asian or Asian British (*n* = 1). All reported to own at least one social media account. Participants were grouped by school year to ensure they were similar in age and had some familiarity with each other to assist with group dynamics (Adler et al., 2019). There were five focus groups, with one focus group for UK school years 7 (11 to 12‐year‐olds), 8 (12‐ to 13‐year‐olds), and 9 (13‐ to 14‐year‐olds), and two focus groups for year 10 (14‐ to 15‐year‐olds). The focus groups had six to eight participants in each and lasted between 50:38–57:42 min.

Ethical approval was granted through the authors' university Research Ethics Committee. A group of participants from each year group with parental consent was asked to participate. All participants provided informed consent at the start of the study and were verbally reminded about their right to withdraw at any time by the lead researcher.

### Procedure

The procedure of this study was conducted in accordance with the COREQ guidelines (Tong et al., 2007). The focus groups were conducted by the lead researcher (female, 23 years old) and took place in June 2022. The lead researcher had experience in facilitating focus groups, conducting qualitative analyses, and completed qualitative methodology training before the start of data collection. At the start of the focus group, the participants were told that the lead researcher's role was an alumnus of the school, with an interest in adolescent social media use.

The focus groups were conducted by the lead researcher and took place at the participants' school during school hours. Participants read the information sheet and completed consent forms before completing a short questionnaire, including basic demographic questions, followed by questions on which social media sites they used and the frequency that they used different reactions. The researcher explained that participants were to focus on ‘reactions’ and explained that this might be ‘liking’, ‘commenting’, ‘sharing’, or other communication tools. The focus groups were audio recorded using a digital recording device. The researcher used a semi‐structured question schedule to guide the discussion and asked prompt questions to encourage further discussion. At the end of the discussion, participants were debriefed.

### Design and analysis

A focus group design was chosen to encourage greater elaboration and discussion of the topics and questions (Heary & Hennessy, 2006). The focus groups were semi‐structured, with the researcher using a question schedule designed to be centred around the key aims of this research study while making allowances for any unforeseen topics that arose as part of the discussion. Prior to data collection, five adolescents (14–17 years old) who did not take part in the focus groups provided feedback on the questionnaire schedule to ensure appropriate wording for the target age groups.

The focus group schedule was designed as a set of questions and prompts that, taken together, allowed the researchers to identify themes on what motivates adolescents to use reactions and how they use reactions to communicate with others. The focus group began with the researcher setting the context with an open question related to what they found to be the best and worst thing about social media. Following this, they were asked about their intentions when they decide to react to a social media post, as well as how they think and feel when they receive reactions from other people. Within these areas, participants were prompted with questions surrounding how individual differences may be involved in how people interpret reactions (e.g., differences for individuals with mental health concerns). Lastly, the researcher asked participants about the new reaction features on social media (e.g., hiding the number of likes on posts), as well as what they would like to change about social media.

Focus groups were transcribed verbatim by the lead researcher and analysed using the six stages of Thematic Analysis (Braun & Clarke, 2006). During transcription, the transcripts were anonymized by removing identifying information but retaining information on their participant number (P) and their age. Step 1: the researcher listened to the audio recordings and read the written transcripts multiple times to become familiar with the data. Step 2: the transcripts were collected to form initial codes across the whole data set using NVivo 12 software (e.g., the statement “when you're reading like a comment, like you can't really tell, like, what way they mean it like in a really serious way or in like a joking way” would relate to the code ‘misinterpretation of reactions’). Step 3: the different codes were organized and combined to create overarching themes that summarize the data in relation to the research question. Step 4: the themes were reviewed and refined to establish validity and to ensure that the themes relate to the coded extracts and the entire data set. Step 5: the themes were given names and descriptions, and a thematic map was developed. Step 6: the researcher gathered extracts from the data set to accurately represent the themes and sub‐themes identified and contextualized the findings in relation to psychological literature surrounding the links between social media reactions, mental health, and the presentation of the self online. The themes identified were analysed and refined through repetition of these phases to ensure validity and consistency (Braun & Clarke, 2013).

Anonymized focus group transcripts, the questionnaire schedule, and supplementary materials can be accessed via the OSF page (https://osf.io/h7jqu/?view_only=b1267d037ce54fb7aad03b2e2c8b5645).

## RESULTS

### Usage of social media apps

To set the scene and understand adolescents' social media use, prior to the focus group discussion, adolescents were asked to complete a short questionnaire that had them reflect on their social media use, including what platforms they use and their current use of reactions. Participants reported using YouTube the most frequently, followed by TikTok, Snapchat, and Instagram (see supplementary materials). Participants also reported how often they used the different reaction functions on social media on a 5‐point Likert scale (see Table 1), with the most reported reaction being the emotive reaction. In addition to set questions, participants noted that they used ‘follow’, ‘favourite’, and ‘dislike’ on social media as a form of communication.

**TABLE 1:** Frequency of social media reaction use across social media platforms within the participant sample (*N* = 34).

Reaction	Never	Rarely	Sometimes	Often	Always
Emotive reaction (Like, Laugh, Care, Love, Wow, Sad, Angry)	2	7	12	13	0
Comments	9	10	13	2	0
Shares	6	13	13	2	0
Save	7	8	13	5	1

### Thematic analysis

**FIGURE 1:**
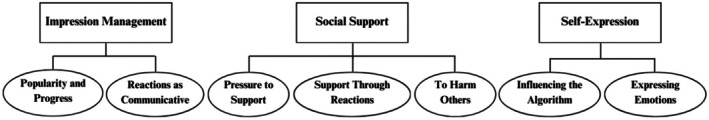
A summary of the key themes from thematic analysis (square) and subthemes (oval).

Three key themes were identified in relation to adolescents' perceptions of the communication intentions of using (and not using) reactions as online interpersonal feedback. The themes were impression management, social support, and self‐expression (see Figure 1 for a thematic map for the themes and sub‐themes).

#### Impression management

Adolescents perceived that reactions on social media are crucial for the formation and maintenance of their impressions online. Understanding how others perceive them online was important for their decision‐making processes regarding when to use a reaction and the circumstances under which they should not react to social media posts. There were two sub‐themes that were identified: (1) popularity and progress, and (2) reactions as communicative.

##### Popularity and progress

Receiving lots of likes and positive comments was viewed as a symbol of popularity by adolescents. If a post received more likes and comments than previous posts, it would be recognized as an indicator that they were making progress in their social standing. This was thought to be directly linked to positive outcomes, such as happiness and accomplishment: “…if you if someone reacts to your post in a positive way, you get uplifted and feel motivated to do like the same thing” (P14, 14 years). Participants explained that some of their peers' motivation to post on social media was to gain gratification in the form of reactions and that receiving many likes and comments made their peers feel validated by others. If a post was to receive fewer likes than they expected or fewer than previous posts, then they believed this would result in negative outcomes, such as confusion, sadness, and disappointment: “I think it wouldn't make you feel as good about yourself if you got less [likes]. If you want more, you'd go back and stuff ‘cause you think you might achieve more ‘cause you got more likes and stuff” (P34, 13 years). Comments were viewed to be more valuable than likes, as a comment was seen as a greater compliment, and it signified that the individual had gone to more effort to express their opinion. As explained by P10 (14 years), “It's more exciting if it's a comment”; however, for comments to be valuable, they should be positively worded “I would rather get a comment but obviously a positive comment” (P30, 13 years).

Participants explained that the number of reactions they received on posts would influence their posting behaviour in the future. It was suggested that they would only post content that received the highest number of likes, even if they wanted to post something else. Some participants commented that they would delete posts if they received negative comments or had fewer likes than expected: “If I like looked back on the post and realised why it got less likes I would actually delete it” (P15, 14 years). However, this appeared to be more important for older adolescents, as some of the younger participants explained that social media interactions were more focused on content enjoyment, as opposed to the number of reactions. For example, P8 (12 years) explained that “sometimes you can post like funny stuff” to which P5 (12 years) added “some people might post for like fun”.

The number of reactions was used as a basis for social comparison between social groups, with suggestions that this comparison led to feelings of jealousy and low self‐esteem: “if I saw somebody else got more comments who's like the same age as me in the same year, it would then make me compare myself to them and be like, oh I'm not as good as them” (P18, 15 years). Despite these comments, when the researcher introduced the feature on Instagram and Facebook that would allow users to hide the number of likes on their posts and on others' posts, no participant expressed an interest in using this function as this would prevent them and others seeing their success or popularity. As explained by P31 (13 years): “Because if you hide your likes and you don't really, can't really see how well you've done. It's kind of like hiding your success from yourself”.

Interestingly, in contrast to findings for older participants, younger participants appeared to have less focus on comparing reactions compared to their older peers. When asked if they compare the numbers of likes to their peers, P7 (12 years) explained “[if someone received more likes] I'd just be like oh good for them or something like that … I wouldn't be like jealous. I'd be like oh well done”. In the same focus group, P8 (12 years) said, “you don't need to be jealous ‘cause they're making something different, so obviously they're gonna like reacted to differently”.

##### Reactions as communicative

Adolescents understood that they used reactions to communicate with others and to exchange information. However, they expressed great concern regarding the potential for misinterpretation of reactions; it was believed that misinterpretation could lead to concerns about negative evaluation from others. Adolescents preferred to give other users likes over comments, as likes were perceived to always be a positive interaction, whereas the text‐based nature of comments meant that they may be misunderstood by others and read differently than intended: “I've got like really bad social anxiety so sometimes I over think of how people might react to a comment even if it is overwhelmingly positive, it's like liking it's easier then it they know you like it, you just move on” (P19, 15 years). Participants did not like to be the first person to react to posts from people they were not friends with: “if it was someone I didn't like I probably wouldn't be the first to ‘like’” (P34, 13 years). P31 (13 years) did not comment first as they are concerned about the wording of comments:

“I'm not sure ‘cause I wouldn't really comment, just'cause … I don't know what to write. And then if I write something then that's gonna be taken the wrong way. So just trying not to hurt someone with a comment that you don't mean harm with. It's ‘cause unlike when you actually have a face to face conversation comments can be taken anyway,’cause you don't know how they're saying it”.

In addition to concerns of misinterpretation, accidentally liking photos provoked feelings of anxiety for adolescents as they believed that this would also negatively affect how others perceived them online: “if you [accidently] like something that someone posted a while ago, then they'll sort of know that you've been to or been looking at their profile stuff so it might be a bit weird” (P13, 14 years). Accidently liking a photo was concerning to the adolescents, as this would have negative implications for their digital self: “if you accidentally like on their video…if you can't unlike it, then they may think you're friends with them when you're not” (P5, 11 years). Although adolescents expressed that they would expect their peers to return the gesture of reacting to their content, “[I'd feel] annoyed … And I'd tell them … Because if I did it for them, then maybe they should do it back?” (P15, 14 years), they would not reciprocate reactions intentionally as this may negatively affect their digital self‐presentation “I just feel like it's a bit weird if you go on their account just after they've, they've liked you … ‘cause it kind of feels kind of stalkerish” (P31, 13 years).

Participants also believed that those with poor mental health would also be more likely to experience magnification when interpreting reactions from others: “If you're posting something and you've got like bad mental health, I feel like you take any reaction more extremely like sometimes if it's a positive reaction we're going to take it way better. But if it's a negative reaction it will take it way worse” (P14, 14 years). They explained that those with anxiety are also more likely to ruminate over comments and interpret ambiguous comments negatively: ‘cause some people aren't in good places when it comes to mental health so when you comment something that isn't doesn't seem bad, so doesn't seem bad to someone who is in a good state of mental health. But to the first person with a bad state of mental health, it's going to feel like a kick to the face’ (P31, 13 years). Furthermore, participants thought that if someone with poor mental health received fewer reactions, it would impact their future social media behaviour: “you might stop doing your content because you don't have as much likes as you want or you make more content to try and get more likes” (P4, 11 years).

#### Social support

The use of reactions on social media was seen as a key part of providing and receiving social support from others online, but they were also seen as a method to withhold support or to harm others. Participants described that if they received a ‘like’ or a positive comment, this was a clear indication that the person supported them or endorsed the content within the post. Participants perceived that receiving support from others influenced self‐esteem, confidence, and positive emotions, such as accomplishment and happiness. Three subthemes were identified, including (1) showing support through reactions, (2) pressure to show support, and (3) using reactions to harm others.

##### Showing support through reactions

Participants expressed that reactions were used to support others. This involved liking posts even if they did not enjoy the post, as they want to show the creator of the post that they support them: “[likes are] a sure sign that they support you and I mean it might make you feel proud and feel confident about yourself” (P26, 15 years). This support was typically provided and received within friendship groups, but adolescents also explained that they would use reactions to support the growth of an ‘influencers’ page on social media: “I normally use liking quite a lot ‘cause um like I, I follow creators that I like, and even if I don't like the video, I'd like them to support them kind of” (P2, 12 years). Another way that they showed support to others is by sharing posts that others would enjoy, and this in turn would invite a reciprocal like and increase social connection: “I just tag my friends wanting them to find it funny as well and they'll tag me as well” (P6, 12 years).

##### Pressure to show support

Adolescents appeared to have norms and expectations surrounding showing social support to their peer groups. Participants felt obligated to react to posts that were from individuals within their social group and feared negative consequences, such as causing arguments or being ostracized from the group, if they did not adhere to the social norm: “Some people, like can force you to like it, so you can't really say no” (P6, 12 years). The pressure to support others may lead to ingenuine comments and compliments: “normally you have to comment like ‘you're so pretty’ and stuff…It's cause, I don't know, it's just out of fear… And even if you don't think it's like pretty or anything, like you can still comment” (P30, 13 years). If participants were tagged in a post by their friends, they felt that they needed to acknowledge this by liking and replying to the comment, even if they did not like the post: “If my friend has tagged me in something, and I just don't enjoy at all and they find it funny I'll probably put the laughing emoji or anything just to like not hurt their feelings” (P11, 14 years).

For younger participants, there was less social pressure to provide ingenuine interpersonal feedback to peers. For example, when asked about their reasons for liking posts, P7 (12 years) explained even if someone expected them to like something, “if [a post contained] something you don't agree with and I wouldn't like it”. P6 (12 years) agreed with P7: “if it's like something you enjoy then you would like it back but if it's something about someone then I probably wouldn't like it”.

##### Using reactions to harm others

Reactions were also used to intentionally not provide support to others; adolescents described how reactions can be used to cause harm to others. Inflicting passive harm was described as withholding reactions from others to prevent the positive outcomes associated with receiving lots of likes and comments, such as accomplishment, happiness, and increased self‐esteem: “If you're like slightly in an argument with someone, and you know that, and you've noticed that they haven't commented on your post, and they haven't liked it … they're doing that to like get back at me or something” (P17, 15 years).

Reactions can be used to cause active harm to others by posting negative comments to embarrass others and to cause the individual to feel negative emotions. Participants explained that bullying is more likely to take place in the comments of posts as opposed to direct messages, as this causes more public humiliation: “Um if you (have) posted a video yourself, sometimes people comment quite nasty things. Like tagging their friends and like saying stuff about you” (P6, 12 years). Cyberbullying within the comments was mentioned across the age groups: “I think comments play a big role in [cyberbullying]. If somebody comments something negative [and] you're being cyber bullied by someone, and they comment something negative … that's also gonna knock them down” (P17, 15 years). P27 (15 years) said, “bad comments [were the worst part of social media]…They're just making fun of like a video you made or something”.

#### Self‐expression within social media

For adolescents, using reactions on social media went beyond communicating and sharing information with others; they also explained that using reactions was a way to manage and maintain the self while online. There were two subthemes identified: (1) influencing the algorithm, and (2) expressing emotions.

##### Influencing the algorithm

Participants were aware of the algorithm on social media and used reactions to encourage similar content to appear again on their feed. It was explained that using reactions in this way made social media more personalized and therefore more enjoyable: “I just like stuff, something I which I think like I want to see more of. If you like it you are most likely to see more of them” (P8, 12 years). This was echoed in another focus group where P20 (15 years) explained, “[Liking images] customises your feed so um you know that when you click on the app, there's stuff that you're gonna like on there”. When speaking about the algorithm, P32 (13 years) explained that using the algorithm in this way can also prevent negative content from appearing on their social media feed: “if you [like] more the good stuff, you won't really get to look at that bad stuff on it”.

##### Expressing emotions

Furthermore, adolescents portrayed the self online by using reactions to convey their emotions towards the content on social media. Likes were used to show that they enjoyed the content; they might have thought it was funny, relatable, or they agreed with the opinions shared in the post: “you could like if it was like funny or like scary or sad or like anything” (P30, 13 years). Comments were used to share their specific opinions towards the content of the post: “I do think that people also might post to share opinions about certain things and certain topics, but it depends on every single person” (P25, 14 years).

When asked about the new features that have been introduced to social media (e.g., the new feature on Instagram to remove the number of likes), participants across the focus groups explained that they understand that some people might use this option if they want to post “…to make them feel better about themselves so they don't really care about likes” (P11, 14 years), or “if somebody gets down on themselves about the amount of likes they get, turning them off could be positive” (P25, 15 years). However, none of the participants in this study said that they would use this function themselves: “it's useless for me” (P14, 14 years). In fact, some commented on how this function could negatively affect them: “I think it's really also negative. Because you won't know like really, how people are reacting to this. It's also actually good to have a negative to stuff as well” (P19, 15 years), “Because if you hide your likes and you don't really, can't really see how well you've done. It's kind of like hiding your success from yourself” (P31, 13 years).

### Would adolescents change anything about social media platforms?

At the end of the focus groups, participants were asked what they would like changed or improved on social media platforms. Some participants explained that they wanted to see stricter algorithms to control negative content: “take down things that they feel that is like offensive or harmful to someone else” (P7, 12 years), “any sort of negativity shouldn't regularly be tolerated” (P10, 14 years). However, across the focus groups, many participants explained that they do not want anything to change, especially with the current like and comment functions. For example, in focus group 2, P13, P10, and P14, aged 14, explained they wanted to keep likes and comments in future apps, which was mirrored in and in focus group 4 from P26 (15 years), with P24 (15 years) adding, “I would keep them because … people are using [likes] to moderate themselves”.

## DISCUSSION

This study explored adolescents' perceptions of communicative intentions in interpersonal feedback on social media, specifically using or not using reactions and how reactions may impact the recipients' thoughts and feelings. There was a focus on specific scenarios when it is appropriate to use reactions or not and on how the use of reactions can have implications for individuals' emotions, relationships with others, and future behaviour on social media. The present study identified three overarching themes: impression management (with the subthemes of ‘popularity and progress’ and ‘reactions as communicative’), social support (with the subthemes of ‘showing support through reactions’, ‘pressure to support’, and ‘using reactions to harm others’), and self‐expression (with the subthemes of ‘influencing the algorithm’ and ‘expressing emotions’). Each of these themes, with the inclusion of their subthemes, will be outlined in turn.

### Impression management

Similar to the dual‐factor model proposed by Nadkarni and Hofmann (2012), this study indicated that adolescents use social media reactions to create and manage impressions online. Reactions, in particular ‘likes’, are used as social feedback from peers on the self that is presented on the social media post (Schlosser, 2020). The number of likes on a post provides quantifiable feedback to adolescents as to whether this version of the self has been accepted or rejected. The feedback allows adolescents to try out various selves on social media and is likely to influence the identity consolidation online (Jong & Drummond, 2016; Michikyan et al., 2015).

Older adolescents in our participant groups appeared more aware of the links between the use of reactions and self‐presentation, whereas younger adolescents focused on the enjoyment of online platforms. As highlighted earlier, as individuals move through adolescence, they become increasingly concerned with social evaluation from peers (Watling & Banerjee, 2007) and are increasingly likely to use self‐presentation to successfully manage impressions (Sarita & Suleeman, 2017). The age differences observed within the findings might be related to the differences in social media experience. Children in early adolescence are just beginning to use social media, whereas older adolescents have had more experience using social media, so they may have a greater focus on creating and managing an online profile. For example, watching YouTube videos or messaging friends on WhatsApp is more common for children under 12 years, while children over 12 years are regularly using social media platforms such as TikTok (Ofcom, 2023). This work highlights the importance of exploring experiences of 11‐ to 15‐year‐olds, leaving questions around the breadth of experiences to be answered in future work.

Although adolescents commented on the importance of receiving likes on their posts, positive comments were preferred as they were seen as more valuable. This could be due to likes being seen as common and automatic, whereas comments show that the user has invested time and effort to comment as well as provide personal social feedback. This finding contradicts some previous work with adolescents, where young people put more of an emphasis on the number of likes, opposed to comments, as likes served as a quantitative measure to compare themselves against their peers (Chua & Chang, 2016). Future research may explore these differences in more detail by experimentally examining attention to likes and comments on social media.

Furthermore, the online interpersonal feedback that adolescents receive is more public than in‐person interpersonal feedback (Nesi et al., 2018), allowing for peers to view and endorse positive and negative feedback (e.g., by liking comments). Adolescents are aware that their social media posts and feedback are public; therefore, they are more likely to take more care over refining the self that is presented (Tice et al., 1995). This finding builds on research by Throuvala et al. (2019), where adolescents reported implicit and explicit rules towards specific social media sites (e.g., maintaining streaks on Snapchat). This was highlighted in the present study as participants commented on taking time to perfect images and changing their social media content to produce the highest number of reactions.

Linking to impression management as a key motivator for the use of reactions, participants also explained that when they accidentally ‘liked’ a post, some reported feeling elevated anxiety; the ‘like’ looks like they have endorsed something that contradicted their values or the impression they are trying to convey on social media. If the liked post was not recent, adolescents believed that this would negatively impact their peers' impression of them. An unintentional violation of a social norm, such as accidental liking, is likely to lead to embarrassment, especially for individuals with higher levels of social anxiety (Bas‐Hoogendam et al., 2018).

As reactions were perceived as a symbol of popularity, the number of likes and comments was reported to be used as a basis for social comparison between peers. This finding adds to previous social media research indicating that social comparison on social media goes beyond comparison of the content of posts (i.e., body image; Fardouly et al., 2015) but shows that adolescents are comparing the number of reactions they receive to that of their peers. Due to the permanency of social media feedback, this provides adolescents with the opportunity to revisit and ruminate over the comparisons of the number of reactions or wording of comments. This goes beyond the initial scope of this study exploring the perceptions and motivations of reaction use on social media, but instead begins to explore the positive and negative consequences of reactions, and how adolescences perceive these.

### Social support

Building social capital online, social connectedness, and gaining access to speciality groups are seen as benefits of social media (Hayes et al., 2022; Selkie et al., 2020). However, this study found that in addition to providing and receiving support from others, adolescents are also using reactions to withhold support from others. This can lead to positive and negative outcomes, as reflected in a review by Allen et al. (2014), where social media helps fulfil young people's need to belong through social connectedness and social interaction but also can make users feel more vulnerable to cyberostracism and loneliness.

Adolescents expressed that online reactions, in particular comments, are used to purposefully harm another person online. It was consistently highlighted that negative comments were used as a tool by cyberbullies to publicly humiliate others. This finding aligned with cyberbullying literature that indicates that cyberbullying occurs both in private messages and in online, public comments (Calvete et al., 2010).

In exploring how adolescents use reactions, it was identified that adolescents felt anxious about keeping up to date with social media posts as there was a need to ensure that they met the social expectations for reacting to social media posts (e.g., ensuring that they like and comment on a friend's post within a certain time frame). This finding is reflected in Jong and Drummond (2016), by adolescent girls showing a desire to obtain immediate feedback on their social media posts, which were associated with exhilaration (when feedback was immediate) and sadness (when feedback was delayed). In fact, our participants recognized that the pressure to react to posts can lead to ingenuine social support to their peers. The receipt of a quick reaction from a peer can be seen as support, but it also may not reflect their true ‘reaction’ to the social media post. This raises questions for future work in considering how a social media user knows when a response is genuine.

### Self‐expression

Social media sites have traditionally been centred around sharing who you are and what you like with your friends and family. Yet, in this study, there was more discussion around using reactions to allow the user to express themselves and to influence what is subsequently shown on their feed. This is an interesting finding, as it appears that adolescents' motivation for using reactions appears to be focused on self‐expression and personal gain. Research has previously identified that authentic self‐expression and identity experimentation on social media are key benefits to well‐being (Bailey et al., 2020). This was also reflected in this study as adolescents found pleasure in using the social media algorithm to tailor their experience. It is important to note that the personal gain of being able to express the self through manipulating the algorithms can have negative consequences if adolescents react to potentially harmful content (e.g., posts that promote risky behaviours or self‐harm), which is then more likely to frequently appear on their social media feed.

### Mental health

Within the focus groups, adolescents were asked to reflect specifically on how those with mental health difficulties may experience online. It was interesting that responses resulted in discussions about motivations and perceptions (interpretations) of social media reactions across a variety of themes. The findings show that there was an understanding from adolescents that individuals may experience social media differently depending on their mental health. Adolescents were aware that those with poorer mental health are more likely to focus on social media reactions. It was believed that their peers who are more anxious or depressed are more likely to notice a change in the number of reactions received and engage in rumination over ambiguous or negative comments. This is supported by previous research that has shown that individuals whose self‐worth is dependent on social media feedback are at greater risk of poorer psychological well‐being (Sabik et al., 2020). Our findings also relate to literature exploring attentional biases on social media. Research has indicated that attentional bias towards social media is correlated to problematic social media use (Zhao et al., 2022), with attentional biases also mediating the association between social media addiction and depression in adolescents (Xiao et al., 2022). Future studies may explore differences in the attention patterns of adolescents with poorer mental health when they are using social media.

### Implications

The findings from this study demonstrate that adolescents are aware of social norms around the use of reactions, which can lead to feelings of wanting to stay up to date with others' posts. In turn, adolescents may want to use their phones for long periods of time or check their social media accounts frequently. Compulsive checking of social media accounts may lead to adolescents experiencing fear of missing out (FoMO) or nomophobia (fear of having no mobile phone), which may link to problematic or excessive social media use (Franchina et al., 2018; Throuvala et al., 2019). Further research could explore the links between social media reactions and FoMO to understand this relationship further. Moreover, it is interesting to note some of the age‐related differences in reaction use from the 11‐ to 12‐year‐old group, who are newer to social media, compared to adolescents who are 12 years and over, where it is expected that they are learning the social norms for social media engagement. Due to some of the gender differences seen within the use of social media (Svensson et al., 2022), future research may also explore gender differences with reaction use, in addition to the developmental patterns seen in this study.

Our findings support and build upon themes shown in a review on social media perspectives by Popat and Tarrant (2023) by highlighting that adolescents recognize that they use reactions, and for more than just ‘liking’ the image. Adolescents understand that they are reviewing social media reactions on their own posts and on their peers' posts to develop judgements about the individuals' self‐worth, popularity, and belonging. Reactions allow the user to tailor their social media experience to express the self and their emotions. In addition, reactions are used to communicate with others online and to show support; however, due to the strict online social norms surrounding when it is appropriate to use reactions, this can lead to adolescents providing ingenuine support to others. Importantly, in considering the social norms and perceptions of reactions, adolescents report that those with mental health concerns are likely to be concerned about receiving, or not receiving, social media reactions. Secondary school educators and parents should aim to understand the complexity of the social norms associated with reaction tools to better support adolescents whilst they are online.

### Strengths and limitations

The use of focus groups allowed for more discussion between peers, for points to build on one another, and to understand if there was widespread agreement across the group. However, we recognize that some adolescents may have felt uneasy sharing details of negative experiences of social media. If research wished to explore more sensitive topics around the negative use of reactions (e.g., experiences of cyberbullying), an interview study design would be more appropriate.

It is important to note that the participants in this study were aged 11–15 years, whereas adolescence is thought to extend to 25 years, after brain maturation (Arain et al., 2013). Therefore, whilst this study can comment on the use of reactions between the ages of 11 and 15 years, further research is needed to understand motivations and perceptions across the full adolescent development.

The study indicates that future research should focus on the online activities that young people partake in, and experiences may differ for those with poorer mental health. It would be important to conduct research with different clinical groups to understand their experiences on social media. Future research should aim to build on this study by quantitatively exploring how adolescents' use social media reactions and if this differs for those with mental health concerns, such as high levels of anxiety or depression.

In conclusion, reactions play an important role in adolescents' experience on social media. Adolescents are using reactions for more than expressing that they like a post. Instead, these functions are used to influence the impressions others form of them, give, receive, and withhold social support, and manage and maintain the self. The finding from this study also shows how reactions can influence the mood of young people and how individuals with mental health concerns may interact with these functions differently. This paper highlights an important contribution to the current literature surrounding social media reactions, as it adds to our understanding of the underlying mechanisms of social media use.

## AUTHOR CONTRIBUTIONS

**Gemma Rides:** Conceptualization; methodology; data curation; formal analysis; project administration; resources; writing – original draft; writing – review and editing. **Helen Pote:** Conceptualization; methodology; supervision; validation; writing – review and editing. **Dawn Watling:** Conceptualization; methodology; validation; supervision; writing – review and editing.

## Data Availability

The data that support the findings of this study are openly available in Open Science Framework at http://doi.org/10.17605/OSF.IO/H7JQU.
